# 2821. A Comparison of Empirical Therapy with Cefazolin Versus Ceftriaxone for the Treatment of Complicated Urinary Tract Infections in a Tertiary Care VA Medical Center

**DOI:** 10.1093/ofid/ofad500.2432

**Published:** 2023-11-27

**Authors:** Paola Carcamo, Teri L Hopkins, Linda Yang, Christopher R Frei, Jose Cadena-Zuluaga, Elizabeth Walter

**Affiliations:** South Texas Veterans Health Care System (STVHCS), San Antonio, Texas; South Texas Veterans Health Care System, San Antonio, Texas; South Texas Veterans Health Care System, San Antonio, Texas; University of Texas, San Antonio, Texas; University of Texas health and science center San Antonio, Audie L. Murphy VA Medical Center, San Antonio, Texas; South Texas Veterans Health Care System (STVHCS), San Antonio, Texas

## Abstract

**Background:**

Limited data exist regarding the utilization of a narrow-spectrum agent such as cefazolin for the treatment of complicated urinary tract infections (cUTIs). The objective of this study was to evaluate cefazolin compared with ceftriaxone for the empirical treatment of cUTIs in an inpatient setting.

**Methods:**

We conducted a single center, retrospective cohort study of Veteran patients who received ≥24 hours of cefazolin or ceftriaxone for the empirical treatment of cUTIs between November 1, 2019 and September, 30 2022. The primary outcome was clinical success, defined as resolution of signs and symptoms of infection without re-initiation of antibiotics during hospitalization or relapse, at 30 days after cUTI diagnosis. Secondary outcomes included hospital length of stay and Clostridioides difficile infection (CDI) within 30 days of the end of antibiotic therapy. A multivariate logistic regression analysis was also conducted to identify independent factors associated with 30-day clinical success.

**Results:**

We identified 118 patients (median age, 72 years; 97% men) with cUTI treated with cefazolin (n = 57) or ceftriaxone (n = 61) who met study criteria. Both study groups had similar demographics, although patients treated with ceftriaxone more frequently had subjective fever on admission or nephrolithiasis while cefazolin-treated patients had more urinary catheters or receipt of another antibiotic prior to study drug. Clinical success was achieved in 51 (90%) and 53 (87%) in the cefazolin and ceftriaxone groups, respectively (P = 0.66). Multivariate analysis identified patients with urinary catheters as an independent predictor of decreased clinical success, with an odds ratio at 30 days of 0.21 (95% CI, 0.06-0.75). Patients treated with cefazolin had a significantly longer length of hospital stay (median 0.4 days longer) compared with ceftriaxone-treated patients (P = 0.02). There were no patients in the cefazolin arm and 4 (7%) patients in the ceftriaxone arm who had CDI within 30 days (P = 0.12).

**Conclusion:**

Cefazolin was similar to ceftriaxone for the treatment of cUTI based on the outcome of clinical success. Local susceptibility patterns to urinary pathogens should guide empiric treatment.

**Disclosures:**

**Christopher R. Frei, PharmD, FCCP, BCPS**, AstraZeneca: Grant/Research Support

